# Low temperature plasma promoting fibroblast proliferation by activating the NF-κB pathway and increasing cyclinD1 expression

**DOI:** 10.1038/s41598-017-12043-w

**Published:** 2017-09-15

**Authors:** Jin-Ren Liu, Gui-Min Xu, Xing-Min Shi, Guan-Jun Zhang

**Affiliations:** 1 Environment and Genes Related to Diseases Key Laboratory of Education Ministry, School of Public Health, Xi’an Jiaotong University, Xi’an, Shaanxi 710061 China; 2 State Key Laboratory of Electrical Insulation and Power Equipment, School of Electrical Engineering, Xi’an Jiaotong University, Xi’an, Shaanxi 710049 China

## Abstract

The potential applications of low temperature plasma (LTP) in wound healing have aroused the concern of many researchers. In this study, an argon atmospheric pressure plasma jet was applied to generate LTP for treatment of murine fibroblast cell (L929) cultured *in vitro* to investigate the effect of NF-κB pathway on fibroblast proliferation. The results showed that, compared with the control, L929 cells treated with plasma for less than 20 s had significant increases of proliferation; the productions of intracellular ROS, O_2_^−^ and NO increased with prolongation of LTP treatment time; NF-κB pathway was activated by LTP in a proper dose range, and the expression of cyclinD1 in LTP-treated cells increased with the same trend as cell proliferation. After RNA interference to block p65 expression, with the same treatment time, RNAi-treated cells proliferated more slowly and expressed less cyclinD1 than normal cells. Furthermore, pretreatment with N-acetyl-L-cysteine (NAC) markedly prevented the plasma-induced changes in cells. In conclusion, the proliferation of L929 cells induced by LTP was closely related to NF-κB signaling pathway, which might be activated by appropriate level of intracellular ROS. These novel findings can provide some theoretical reference of LTP inducing cell proliferation and promoting wound healing.

## Introduction

Low temperature plasma (LTP) has been used for sterilization in biomedical fields for several years^1–3^. The novel applications of LTP in wound healing, dental care, dermatological therapy, cancer treatment and so on have aroused great interests among researchers of both plasma physics and biomedicine^4–7^. A particular concern is that an appropriate dose of LTP can be effective in treating various skin wounds, including chronic, acute wounds and burn^4,8,9^, etc. Some research indicate that LTP can significantly reduce bacteria around wounds and potentially stimulate the proliferation of epithelial cells and immune cells^10,11^. Our preliminary studies have showed LTP could induce fibroblast proliferation around the wound in mice^12^. However, the mechanisms of how LTP to induce fibroblast proliferation are still unclear.

Nuclear transcription factor κB (NF-κB) is known to regulate gene expression in host defense, immune response, inflammation, cell proliferation, and cell survival. NF-κB is activated by a series of stimuli including cytokines, growth factors, bacterial products, receptor ligands, viruses, reactive oxygen species (ROS), and ultraviolet (UV)^13,14^. Notably, NF-κB up-regulates the transcription level of cyclinD1, which is a vitally important protein promoting cell cycle transition from G1 to S phase^15^. LTP is composed of complex chemical compositions, such as exited atoms, electrons, ions, free radical, UV, and so on^16^. These active particles can react with cell culture medium and tissues to form reactive oxygen and nitrogen species (RONS). Researchers suggest that RONS play pivotal roles in cell or tissue response to LTP treatment^7^. Therefore, we presume that LTP can induce L929 cell proliferation by activating NF-κB signaling pathway.

In this study, we firstly detected the components of LTP in gas and liquid phase and confirmed that LTP could induce L929 cell proliferation with cell viability assay and cell cycle distribution analysis. Secondly, with fluorescence probes, we observed that after LTP treatment, the intracellular ROS, O_2_^−^ and NO productions increased when the treatment time was prolonged. Thirdly, we detected the expressions of phosphorylated NF-κB p65 (phospho-p65), IκB and translocation of phospho-p65 from cytoplasm into nucleus with Western blotting and immunofluorescence (IF), respectively. It was found that NF-κB pathway was activated by LTP within a proper dose range. Through analyzing the expression of cyclinD1 extracted from LTP-treated cells, we discovered that the changes of cyclinD1 expression had the same trend with cell proliferation. However, pretreatment with N-acetyl-L-cysteine (NAC) markedly prevented the plasma-induced changes described above in cells. Finally, when the NF-κB pathway was blocked with RNA interference, RNAi-treated cells proliferated more slowly and expressed less cyclinD1 than normal cells with the same treatment time. These findings will provide some beneficial support of LTP inducing cell proliferation and promoting wound healing.

## Results

### APPJ device and its optical emission spectra

**Figure 1:**
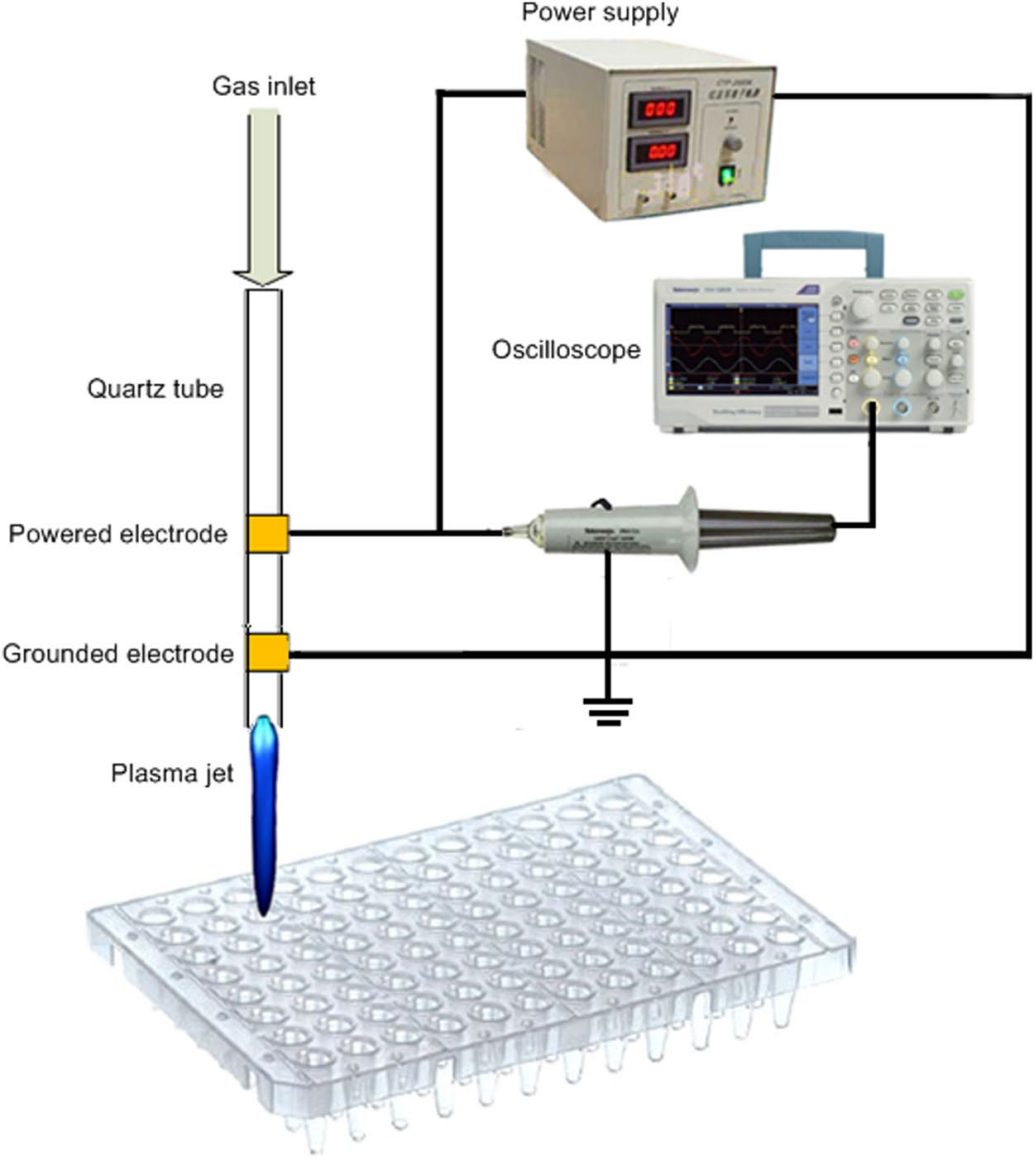
Schematic diagram of the experimental setup.

The plasma source in argon was generated by a co-axial double ring electrodes configuration as described elsewhere^12^. The schematic diagram of the APPJ device is shown in Fig. 1. A hollow quartz tube was used as the barrier dielectric and had inner and outer diameters of 0.2 and 0.4 cm, respectively. The powered electrode and the grounded electrode were 1 cm-wide copper strips wrapped around the tube, and the distance between them was fixed at 1.65 cm. The distance between the grounded electrode and the quartz tube nozzle was 1 cm. The flow rate of working gas was set as 0.5 L/min. An intermediate frequency (~39.5 kHz) sinusoidal resonant power supply (CTP-2000K, Suman Electronics Co. Ltd., Nanjing, China) with a peak voltage of 7 kV was applied to the two electrodes to ignite the plasma discharges. The applied voltage was recorded by a 4-channel digital oscilloscope (Tektronix TDS 4034, Beaverton, OR) through a high voltage probe (Tektronix P6015A). Measured by an alcohol thermometer, the temperature of the plasma jet at the tip of the effluent varied from 25 to 36 °C, similar to room temperature. The emission spectra of the plasma jet were collected by a spectrometer (Mechelle 5000, Andor Technology Ltd., Belfast, UK) together with an intensified charge coupled device (ICCD) (iStar334, Andor Technology Ltd.).

**Figure 2:**
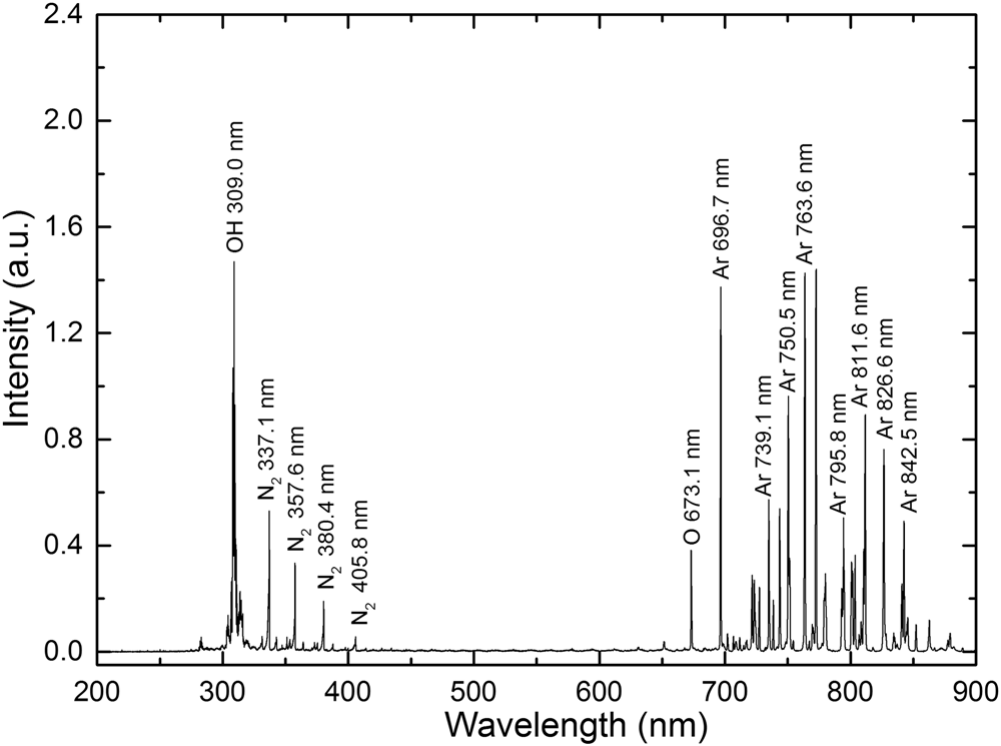
Representative optical emission spectra of the argon APPJ (Ar flow rate 0.5 L/min).

The APPJ emission spectra in the wavelength range of 300–900 nm were captured and the identified lines are shown in Fig. 2. It can be seen that the mainly lines emitted by excited atoms of the feed gas argon were between 680 and 900 nm, indicating that the discharge was dominated by electron impact ionization. Peaks corresponding to N_2_ were measured between 330 and 400 nm and were presumably present due to mixing of the feed gas argon with the surrounding ambient air along the plasma jet. Atomic oxygen line at 673 nm and OH radical at 309 nm were detected, presumably as a result of O_2_ and H_2_O dissociation. Atomic oxygen and OH radical are highly reactive radicals that could play important roles in the potential biomedical applications of plasmas such as bacteria inactivation and wound healing. Furthermore, reactive oxygen and nitrogen species (ROS and RNS) can influence the intracellular environment, possibly by diffusing into cells or by inducing new species within the cells^12^.

### H_2_O_2_, nitrite/nitrate produced in phosphate buffered saline (PBS) and dulbecco’s modified eagle medium (DMEM) without cells

**Figure 3:**
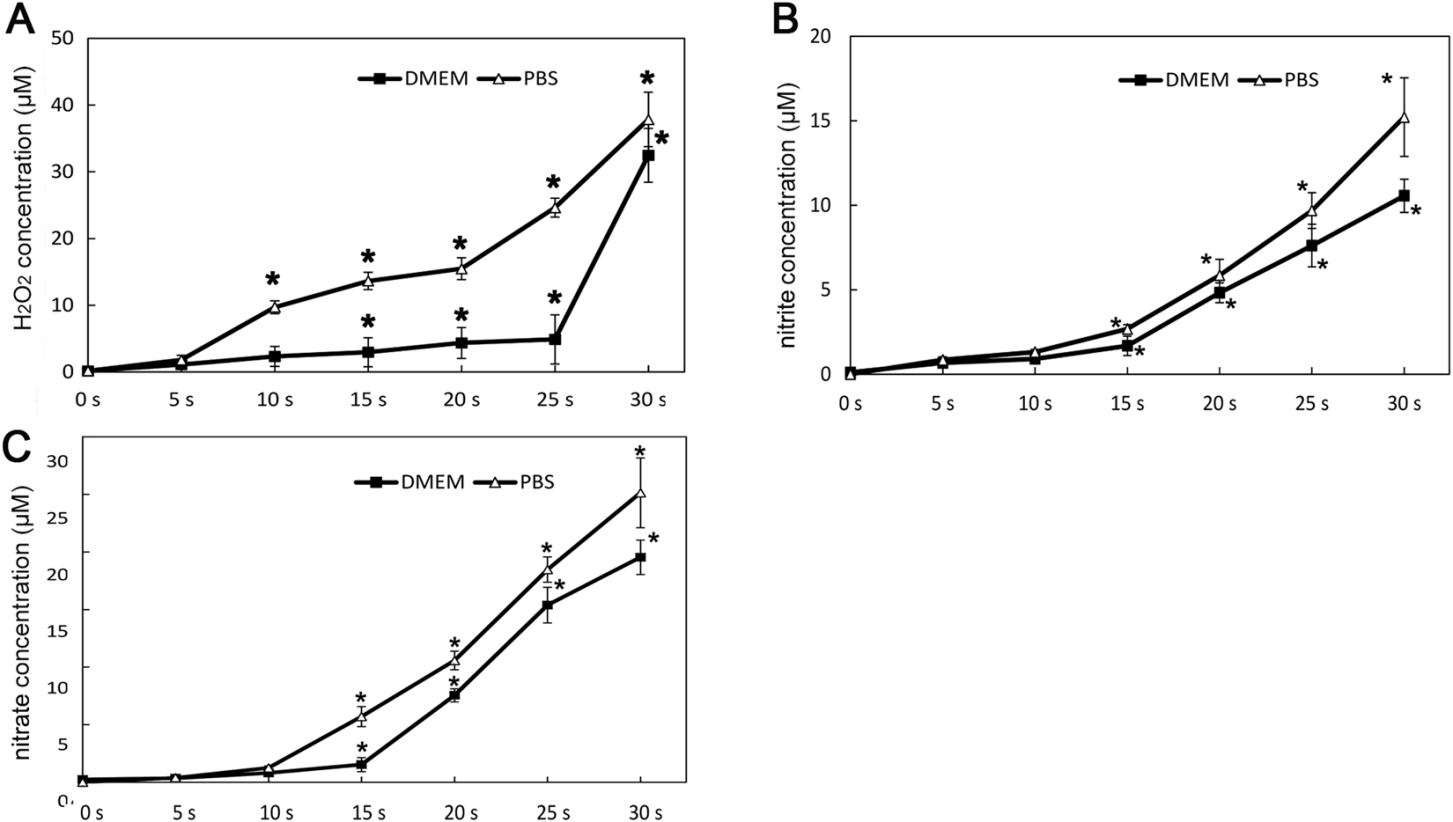
Concentration of H_2_O_2_, nitrite/nitrate in LTP treated PBS and DMEM. 200 μL PBS or DMEM were set to per well in 96-well plates, and exposed to LTP for the indicated peroid of treatment time. (**A**) The concentrations of LTP–induced H_2_O_2_ in PBS or DMEM were determined by using CBA and catalase. (**B**),(**C**) The concentrations of nitrite and nitrate LTP induced in PBS and DMEN were detected with UV-visible absorption spectroscopy. The data are the mean ± SD of three independent experiments.

The concentrations of LTP-induced H_2_O_2_ in PBS and DMEM were detected with coumarin boronic acid (CBA) probe and shown in Fig. 3A. H_2_O_2_ was assayed with and without the catalase by observing the fluorescence of the product, 7-hydroxycoumarin (COH)^17^. It could be seen that H_2_O_2_ was produced in both PBS and DMEM obviously with the prolongation of LTP treatment time and the concentration in DMEM was lower than that in PBS of each groups (p < 0.05). Yan *et al*. found that the components in DMEM such as cysteine and methioine were the main factors that promoted degradation of reactive species including H_2_O_2_^18^.

Besides, to quantify nitrite/nitrate produced in the PBS and DMEM by LTP, 200 μl of PBS or DMEM were exposed to LTP for 0, 5, 10, 15, 20, 25 and 30 s and the amount of nitrite/nitrate were quantified by UV absorption spectroscopy, as described in the Material and Methods section. The results in Fig. 3B,C showed that a time-dependent increase of each compound concentration, and the concentration of nitrite was lower than that of the nitrate. Normally, DMEM contains a certain amount of nitrate. Moreover the reductive components of DMEM including cysteine and serum maintain nitrite/nitrate in a low level. So a less than 10 s LTP did not cause a large change of nitrite/nitrate concentration. With the treatment time was more than 15 s, the accumulated nitrite/nitrate were sufficient to withstand the buffering of DMEM or PBS, resulting for the rapid increase of nitrite/nitrate (p < 0.05).

### Intracellular ROS, O_2_^−^, and NO production in LTP-treated L929 cells

**Figure 4:**
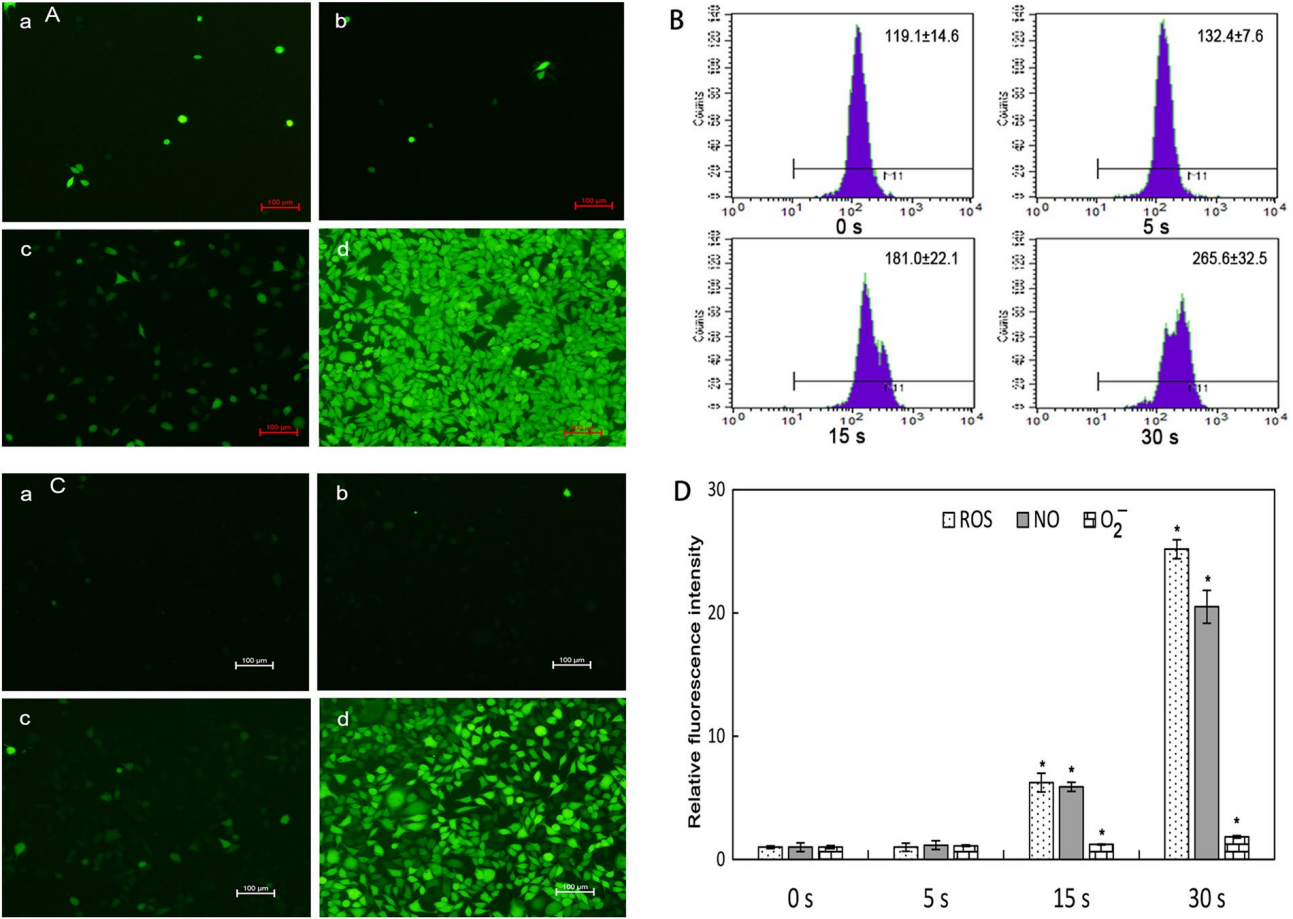
Intracellular RONS productions induced by LTP (*p < 0.05 vs control). LTP-induced intracellular ROS, O_2_^−^ and NO productions in L929 cells were detected after 24 h incubation. (**A**) Intracellular ROS was determined with 2′, 7′-dichlorofluorescin diacetate (DCFH-DA). (**B**) Intracellular O_2_^−^ was determined with dihydroethidium (DHE), which was oxidized by O_2_^−^ and then produced ethidium, which combined with DNA to produce fluorescence. The ethidium fluorescence was detected at 535 nm for excitation and 610 nm for emission with flow cytometry. (**C**) Intracellular NO was determined with 3-Amino, 4-aminomethyl-2′, 7′-difluorescein, diacetate (DAF-FM DA). (**D**) Bar graph showed the relative fluorescence intensity of each type of RONS. Data were calculated three times based on fluorescent intensity. Scan bar is 100 μm.

A series of fluorescence probes were applied to characterize the intracellular oxidative production of LTP-treated L929 cells. The results demonstrated that all these three kinds of intracellular RONS increased gradually when the treatment time was longer than 5 s. The results were shown in Fig. 4A–C, representing ROS, O_2_^−^ and NO, respectively. It could be seen that LTP could induce ROS, O_2_^−^ and NO productions in time-dependent manner. After LTP treatment of 15 s, the cells displayed 6.0, 5.8 and 1.5 folds increase of ROS, NO and O_2_^−^ compared with their controls (p < 0.05). When the treatment time was 30 s, intracellular RONS productions increased rapidly compared with the control, and ROS, NO and O_2_^−^ productions increased 25.2, 20.5 and 2.2 folds, respectively (p < 0.05).

### LTP induced L929 cell proliferation *in vitro*

**Figure 5:**
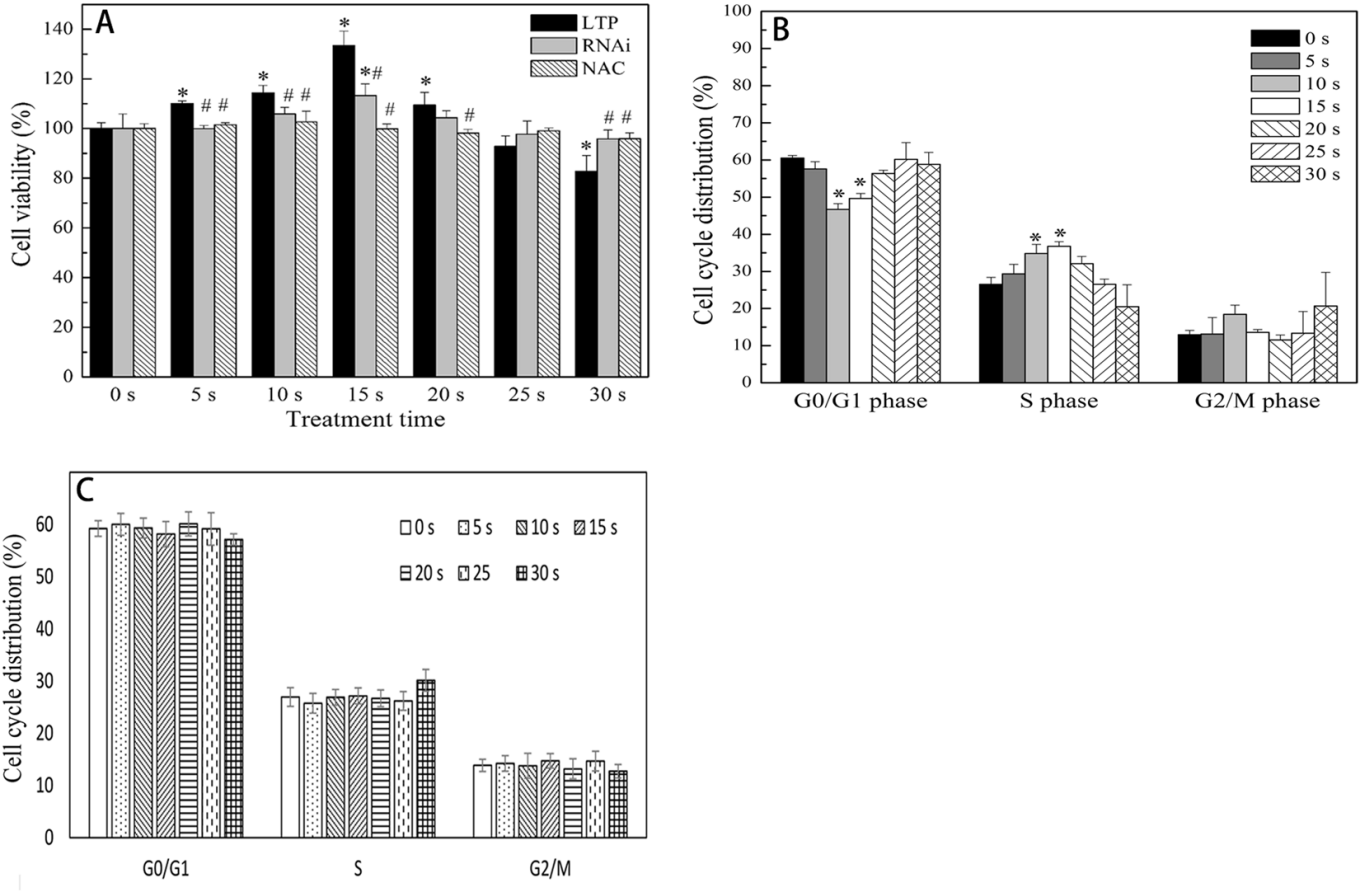
Effects of LTP on L929 cell proliferation and cell cycle distribution. (**A**) Cells were treated with LTP and cell viability was detected with MTT assay after 48 h incubation. Normal cell viability increased at 5, 10 and 15 s and peaked at 15 s. However, when the treatment time was longer than 25 s, cell viability decreased significantly (*p < 0.05 vs control). Viability of p65siRNA-transfected L929 cell increased at 15, 20 s (*p < 0.05 vs control). No significant difference was found in NAC pretreatment cells (*p > 0.05 vs control). Compared with normal cells, viability of p65siRNA-transfected and NAC pretreatment cells decreased at 5, 10 and 15 s and increased at 30 s (^#^p < 0.05 vs normal cell groups). (**B**) LTP-treated cell cycle distribution was detected with flow cytometry after 48 h incubation. More cells transited into S phase from G0/G1 phase at 10 and 15 s compared with the control (*p < 0.05 vs control). (**C**) After pre-treatment L929 cells with NAC for at least 4 h, LTP-treated cell cycle distribution was assayed with flow cytometry after 48 h incubation. No obvious changes had been tested with the treatment time prolongation compared with control. The data are the mean ± SD of three independent experiments.

To verify the effects of LTP on L929 cells, we treated each group of cells with different exposure intervals of 0 s (control group), 5 s, 10 s, 15 s, 20 s, 25 s and 30 s, respectively. After that, we characterized cell proliferation with cell viability and cell cycle distribution. The effects of LTP on cell viability were shown in Fig. 5A. The results indicated that, 5 s, 10 s and 15 s LTP exposure could enhance cell viability significantly compared with 0 s group (p < 0.05), and a peak value at 15 s exposure was observed. However, when the treatment time was longer than 25 s, the OD value decreased gradually with the extension of exposure time (p < 0.05). The flow cytometry results shown in Fig. 5B indicated that a proper LTP exposure dose could promote more cells from G0/G1 phase into S phase. Quantitative analysis showed that the percentage of S phase cells in the control group was (28.41 ± 0.54) %, which significantly increased in the 15 s LTP-treated group (37.02 ± 0.37) %. The ANOVA tests showed that the differences in the distributions of cell cycle among 10 s, 15 s and 0 s groups were statistically significant (p < 0.05). Besides, pretreatment cells with N-acetyl-L-cysteine (NAC), a ROS scavenger, significantly prevented the plasma-induced cell proliferation. (Fig. 5C).

### LTP treatment activated NF-κB pathway

**Figure 6:**
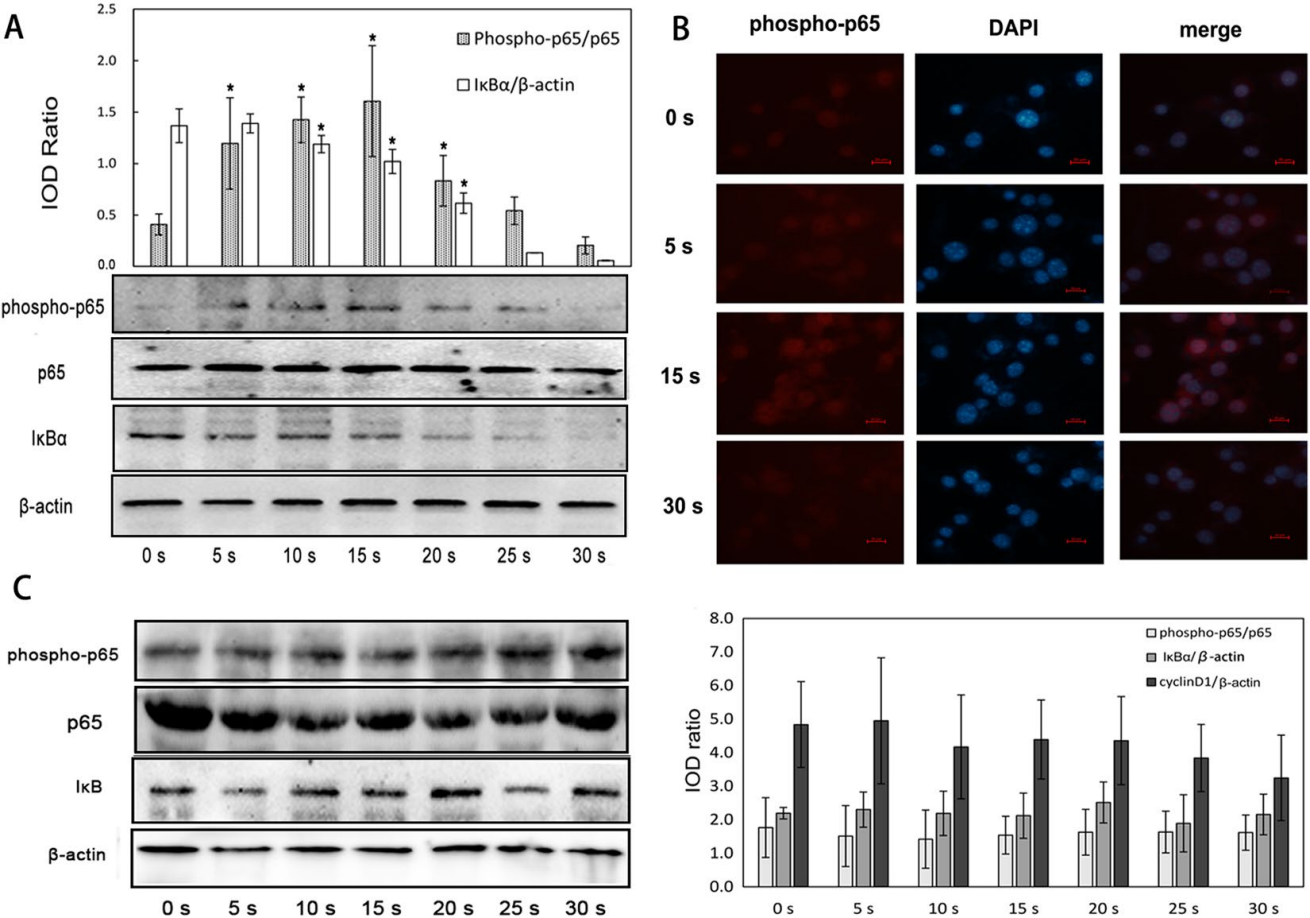
NF-κB pathway activated by LTP (*p < 0.05 vs control). (**A**) Phosphor-p65 and IκBα were detected in the extracts of LTP-treated cells after 48 h incubation. Extracts were separated with SDS-PAGE and analyzed with Western blotting. Expression of total p65 and β-actin served as inner references. The bar figure showed semi-quantitative analysis results. IOD ratio represented the ratio of target proteins (phosphor-p65 and IκBα) to their inner references in each group. (**B**) Phosphor-p65 in the nucleus of LTP-treated cells was detected with IF. The phospho-p65 protein was red, nucleus was blue, and images were digitally merged. Scan bar was 20μm. (**C**) After pre-treatment L929 cells with NAC for at least 4 h, protein were extracted from LTP-treated cells after 48 h incubation and analyzed with western blotting. Expression of total p65 and β-actin served as reference for phospho-p65 and IκBα, respectively. The bar figure showed semi-quantitative analysis results. IOD ratio represented the ratio of target proteins (phosphor-p65 and IκBα) to their inner references in each group.

Previous studies showed that NF-κB pathway could be activated by ROS and other stimulating factors^14^. In addition, LTP reacted with cell culture medium to form RONS and other activated species^7,19^. To determine whether LTP could activate L929 cells *in vitro*, phospho-p65 and IκBα, key molecules involved in the NF-κB pathway were extracted from LTP-treated cells and determined with Western blotting. Besides, the transfer of phospho-p65 from cytoplasm into nucleus was regarded as a significant symbol of NF-κB pathway being activated. So we applied IF staining to analyze phospho-p65 translocation in nucleus. The results indicated that an appropriate dose of LTP could activate NF-κB pathway in L929 cells *in vitro*. It could be seen in Fig. 6A, compared with control groups, in the 5 s, 10 s and 15 s treatment groups, the expression of phospho-p65 increased gradually (p < 0.05). However, it decreased with 30 s treatment (p < 0.05). When the treatment time was longer than 10 s, IκBα expression decreased compared with control (p < 0.05). Figure 6B exhibited the fluorescence images of LTP-treated cells stained with fluorescent dyes, in which nucleus was blue and phospho-p65 was red. These results showed that more phospho-p65 emerged in the nucleus and cytoplasm at 15 s treatment compared with 0 s and 30 s.

Furthermore, 5 mM NAC was used to eliminate LTP induced ROS in cell culture medium, and then phosphor-p65 and IκBα were analyzed with western blotting. The results showed that the expression of phosphor-p65 had no obvious change with the treatment time prolongation compared with total p65. No variations were found in the expression of IκBα in each groups. It was indicated that LTP-induced ROS in medium were closely related to active NF-κB pathway (Fig. 6C).

### CyclinD1 expression in LTP-treated L929 cells

**Figure 7:**
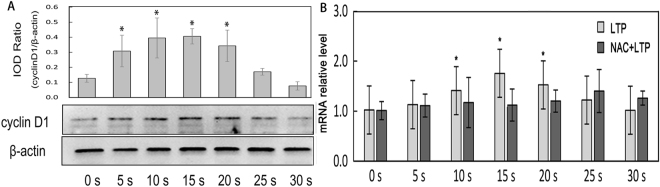
The expression of cyclinD1 in cells (*p < 0.05 vs control). (**A**) CyclinD1 was extracted from LTP-treated and after 48 h incubation, separated with SDS-PAGE, and then analyzed with Western blotting. β-actin served as an inner reference. Semi-quantitative analysis of cyclinD1 was shown in the bar figure. (**B**) The transcription level of cyclinD1 mRNA in LTP alone and NAC+LTP treated cells were detected by q-PCR. β-actin served as an inner reference. 2^−ΔΔCt^ was regard as the level of mRNA transcription level.

As a cell cycle related protein, cyclinD1 plays an important role in G1/S phase, and promotes cells from G1 phase into S phase and commences the DNA replication. We detected cyclinD1 expression with Western blotting, and discovered that the expression of cyclinD1 was related to LTP treatment time (Fig. 7A). CyclinD1 increased significantly from 5 s to 20 s and reached the peak at 15 s. The expression of cyclinD1 had the similar trend with cell viability after LTP treatment. Besides, cyclinD1 mRNA were detected by quantitative RT-PCR at the level of transcription and the results were shown in Fig. 7B. It could be seen that mRNA transcription level of the 15 s group increased 1.76-folds compared to control (p < 0.05). The expression of cyclinD1 in NAC pre-treatment cells was displayed in Fig. 6C. It could be seen that there were no obvious variation about cyclinD1 expression and mRNA transcription (Fig. 7B).

### Cell proliferation, cell cycle distribution and cyclinD1 expression in p65si-transfetced L929 cells treated with LTP

**Figure 8:**
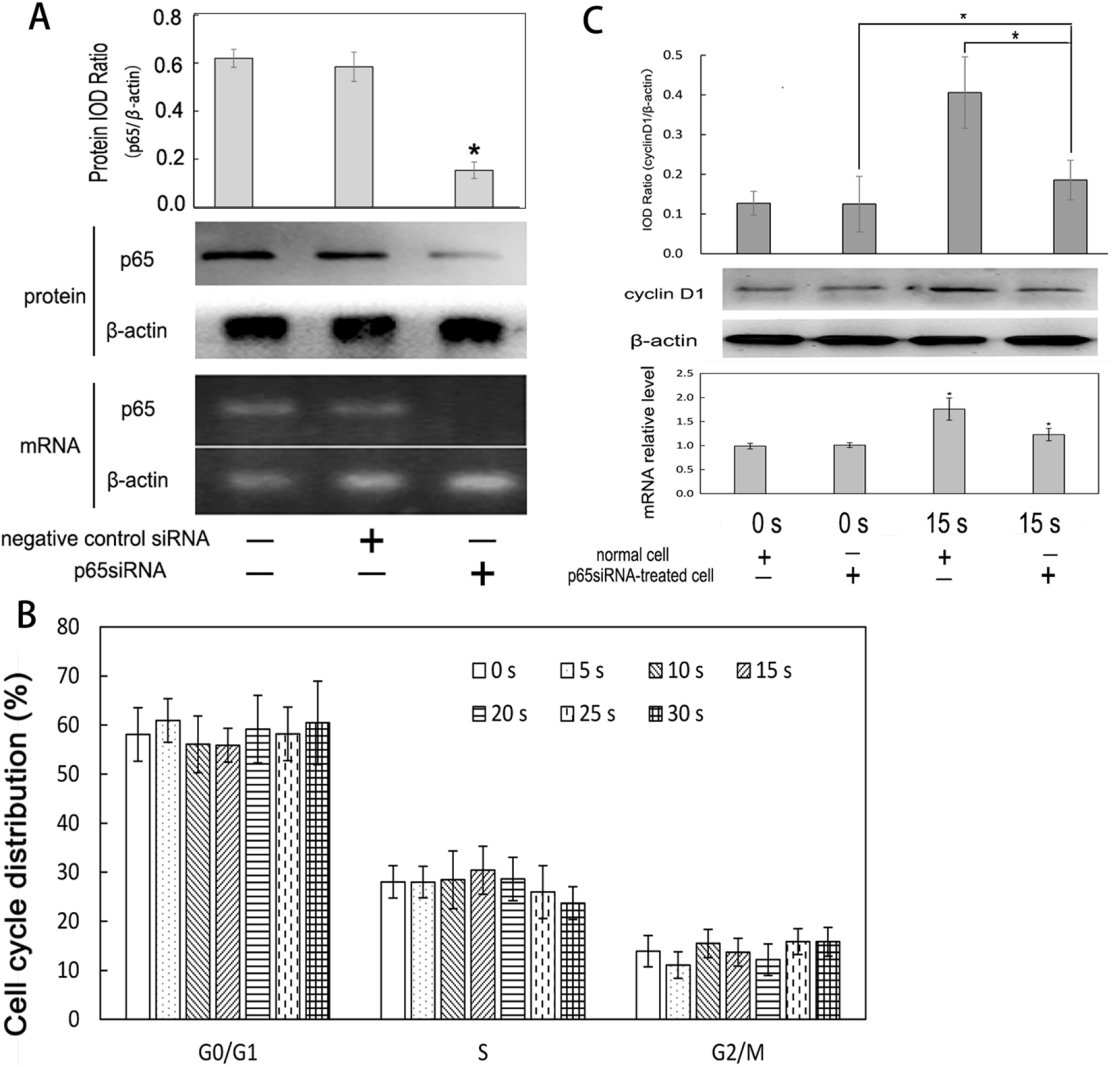
Cell viability, cell cycle distribution and expression of cyclinD1 in p65siRNA-transfected L929 cells (*p < 0.05 vs control). (**A**) The images of mRNA transcription and protein expression. The full-length images were presented in Supplementary Figures 1 and 2. L929 cells were transfected with siRNA oligo and lipofectamine^TM^ 2000. Total RNA was extracted from p65siRNA-transfected cells after 24 h incubation and analyzed with reverse transcriptional PCR. The transcription level of p65 and β-actin mRNA in blank control, negative control and p65siRNA cells were shown in the bottom bar in A. The middle bar in A showed that p65 protein expression was analyzed with Western blotting. The upper bar in A showed that the expression of p65 in p65siRNA-transfected cells was lower than other groups (*p < 0.05). (**B**) Cell cycle distribution of LTP-treated p65siRNA-transfected cells were detected by flow cytometry. (**C**) The images of mRNA transcription and protein expression. The full-length images were presented in Supplementary Figure 3. The protein and mRNA were extracted from p65siRNA-transfected cells and normal cells treated by LTP for difference treatment durations and analyzed by Western blotting and q-real time PCR.

In order to clarify the relationship between NF-κB signal pathway and LTP-induced proliferation, we attempted to construct a blocked NF-κB pathway cell model by RNA interference. We applied specific small interfering RNA (siRNA) to knock down the expression of NF-κB p65 subunit. Figure 8A showed the level of p65 mRNA transcription and protein expression. An inhibition rate (66.87 ± 8.52) % indicated that the blocked-NF-κB signal pathway cell model was constructed successfully.

The same plasma treatment protocol as mentioned previously was used to investigate whether LTP could induce p65siRNA-treated L929 cell proliferation or not. For p65siRNA-transfected cells, the results (Fig. 5A) revealed that cell viability significantly increased at 15 s (p < 0.05). However, cell cycle distribution in Fig. 8B showed that there were no statistically significant differences among the groups (p > 0.05). The data of cell proliferation of both normal and p65siRNA-transfected L929 cells were analyzed. With the same treatment time, the viability of p65siRNA-transfected cells was significantly lower than that of the normal cells at 5 s, 10 s and 15 s (p < 0.05). Though we treated these two types of cells for the same time, the cell proliferation of p65siRNA-transfected cells was significantly inhibited. These findings demonstrated that NF-κB pathway played a critical role in LTP-induced cell proliferation *in vitro*.

LTP-induced expression of cyclinD1 in p65siRNA-transfected cells and normal cells were evaluated. The results (Fig. 8C) showed that with 15 s LTP treatment, p65siRNA-transfected cells had 1.2-folds increase of cyclinD1 mRNA transcription compared with the control (p < 0.05). However, for normal cells, the transcription of cyclinD1 mRNA increased 1.8 folds compared with the control (Fig. 8C). Compared with normal cells (Fig. 8C), p65siRNA-transfected cells had cyclinD1 expression dropped by 45.61% (p < 0.05) with the same treatment time (15 s). These results suggested that LTP-induced cyclinD1 expression was closely related to NF-κB pathway in L929 cells *in vitro*.

## Discussion

Fibroblasts play a key role in the proliferative and remodeling phases of wound healing. The main functions of fibroblasts include synthesis of extracellular matrix (ECM) molecules, release of multiple proteases, such as interstitial collagenases, gelatinase, matrilysin, *et al*., secretion of cytokines and contraction of wound^20,21^. What is noteworthy is that cytokines synthesized by fibroblasts, including bFGF, TGF-β, IL-1, IL-6, TNF-α, endothelin-1 and so on, not only stimulated proliferation of fibroblasts themselves, but also affected other cells in a paracrine fashion^22–24^. Our previous studies revealed that the number of fibroblasts in the tissues around the LTP-treated wound in mice significantly increased^12^. E Sysolyatina *et al*. discovered that LTP promoted fibroblast proliferation and suggested this effect might be related to cell autophagy^25^. Paola Brun *et al*. provided evidence that helium generated plasma promoted proliferation and migration in liver and intestinal fibroblast-like primary cells mainly by increasing intracellular ROS levels and elicited the PPAR-c anti-inflammatory molecular pathway^26^. In this study, we found a similar proliferative effect of LTP on L929 cells. By focusing on the role of NF-κB signaling pathway in LTP-treated cells, LTP activated NF-κB pathway and it association with cell proliferation were revealed.

RONS include superoxide anion (O_2_^−^), hydroxyl radical (OH^•^), hydrogen peroxide (H_2_O_2_), nitric oxide (NO), peroxynitrite (ONOO^−^), and so on, which derived not only from cellular metabolism but from external environment. It is important to maintain a dynamic balance of RONS in organisms^27^. Previous studies had proved that there was a dose-response relationship between RONS and cell functions. A normal level of RONS played important roles in organism signal transductions, including activating transcription factors (NF-κB)^14^, regulating phosphorylation and dephosphorylation of target proteins (MAPK), and maintaining calcium signaling channel^28,29^. However, higher level of RONS damaged nucleic acids, proteins, fatty acids and other molecules and induced cell apoptosis and even caused necrosis^30–32^. According to current researches, LTP-induced RONS were generated, transferred or interacted in culture medium. Furthermore, these extracellular RONS could enter cells possibly by diffusing and inducing new species of intracellular production^7^. O_2_^−^, with strong oxidization and very short half-life, dismutated to hydrogen peroxide (H_2_O_2_) and oxygen by either enzyme SOD or no-enzymatic reaction system. Protonation of O_2_^−^ produced hydroperoxyl radical (H_2_O^−^), which subsequently caused lipid peroxidation and damaged biological membranes^33,34^. NO had diversified effects on cytostatics, chemotaxis, and vasodilation during early stage of wound healing, and it also regulated the proliferation, differentiation and apoptosis of cells involved in wound healing^35^. However, NO had negative effects: excessive or disordered production would cause a series of adverse outcomes including free radical damage, chronic inflammation *et al*.^36,37^. Our study observed that the productions of intracellular ROS, O_2_^−^ and NO increased with prolongation of LTP treatment time. It may be the reason why the fibroblast proliferation, NF-κB pathway-related proteins, and cell cycle-related protein increased firstly and then decreased. It was reported that RONS produced extracellularly by plasma may immediately move across the cell membrane by active transport across the membrane, transient opening of pores in the membrane, then interact with amino acids and proteins and finally lead to production of long-lived reactive hydroperoxides or activation of pathways that modify intracellular ROS concentration^38^. In our present study, the intracellular RONS had no significant change, whereas cell proliferation, NF-κB pathway activation occured with 5 s LTP treatment. It could be explained that the intracellular RONS might mainly activate signaling pathway and promote cell proliferation, which led to RONS consumption. Therefore, the intracellular RONS in 5 s group could not accumulate effectively and were no statistically significant compared with control. Furthermore, in present study, NAC was added into cell culture medium to eliminate LTP-evoked ROS. No obvious variations of cell proliferation, cell cycle distribution, phospho-p65 and cyclinD1 expression were observed compared to normal cells. This stategy may provide evidence that LTP-induced ROS play the critical role in cell proliferation.

It should be noted that there is no way to accurately obtain qualitative and quantitative detection results of intracellular free radicals. As a result, the common fluorescent probes such as DHE, DCFH-DA and DAF-FM DA^39^ were used in this study. These probes can only reflect the trend of free radicals variance but not quantify the concentration of free radicals.

Our study also demonstrated that LTP could activate NF-κB signaling pathway. NF-κB, as a familiar transcription factor, can respond to environmental stimuli with specific changes in gene expression. A wide variety of genes are regulated by NF-κB, including encoding cytokines, chemokines, adhesion molecules, acute phase proteins, inducible effector enzymes, antimicrobial peptides^13,40^. NF-κB forms homodimer and heterodimer with such five proteins: RelA (p65), c-Rel, RelB, NF-κB1 (p50), and NF-κB2 (p52), among which p65/p50 complex is the most abundant form and shows a great transcriptional activation. IκBs are bound with the nuclear localization signals (NLS) on NF-κB subunits. As a result, NF-κB is localized to the cytoplasm in an inactive form in unstimulated cells. As the major IκB protein, IκBα has been studied extensively. When cells are stimulated by cytokines, growth factor, oxidative stress, and other stimulus mentioned before, IκB kinases (IKKs) mediate IκBs phosphorylation, and then the protein is degradated by the ubiquitin and proteasome systems. With that an unmasked NLS p65/p50 heterodimers would be phosphorylated by IKK and others on p65 subunits^13,41^. Activated NF-κB translocates to nucleus from cytoplasma and binds at κB sites in the DNA. Our study showed that NF-κB signaling pathway was activated through the decrease of IκBα, increase of phospho-p65 and translocation of phospho-p65 into nucleus after 24 hours of LTP treatment, as shown in Fig. 6.

NF-κB can be activated by oxidant in many types of cells. The activation methods mainly include ROS activating NF-κB signaling pathway by IκBα phosphorylation at serine 32 or 36 and phospherylation IκBα on tyrosine 42 or PEST domain of serine residues^42^ For instance, hydrogen peroxide activated IKKs^43^, and singlet oxygen activates IκBα through an intact tyrosine 42^44^. Additionally, the phospherylation or dephospherylation and oxidation of thiol residues caused by ROS plays a critical role in ROS-induced transformation of the NF-κB signaling pathway^45^. In the previous studies, NO regulated NF-κB activation to inhibit the production of many proinflammatory factors. In normal human peripheral blood mononuclear cells, NO promoted cytoplasmic NF-κB proteins to translocate into nucleus^22^. Other studies with vascular endothelial cells revealed that NO could inhibit NF-κB target genes transcription through either protecting IκBα from proteolytic degradtion or upregulating transcription of IκBα gene^46^. In this study, we detected the crucial biomakers IκBα and phospho-p65 extracted from LTP-treated L929 cells, and the results demonstrated that LTP could activate NF-κB pathway at an appropriate dose. Besides, pretreatment with NAC markedly prevented the plasma-induced variations of cell viability, cell DNA synthesis, NF-κB pathway activation and cyclinD1 expression in cells. We presume that the mechanism is closely related with LTP-induced RONS productions.

As a DNA-binding protein, NF-κB can promote cells from G1 phase to S phase by enhancing expression of cyclinD1^47^. CyclinD1 plays an essential role in G1/S phase checkpoint and DNA synthesis. Normally it associates with cyclin-dependent kinases CDK4 and CDK6, and phosphorylates the retinoblastoma protein (Rb) to release transcription factor E2F in mid-G1 phase^48^. E2F regulates transcription of genes such as CDK1, cyclinA, and cyclinE. Our study revealed that the changes of cyclinD1 expression were similar to the tend of cell proliferation mentioned above.

However, cyclinD1 gene transcription is regulated by not only NF-κB, but also STAT5, Ets1, c-Jun, Otc1 and so on. To ealuate the hypothesis that LTP-induced cyclinD1 increases in connection with NF-κB pathway, we knocked down p65 expression, and subsequently treated these cells with LTP. The results in Fig. 8 showed that cells which blocked NF-κB pathway proliferated slowly with treatment time prolonged and more cells were arrested in G1 phase. With the same dose of LTP, cyclinD1 expression in cells which block NF-κB pathway were significantly decreased than normal cells. So we consider that NF-κB-regulated cyclinD1 expression plays an important role in LTP-promoted L929 cell proliferation *in vitro*.

## Methods

### Cell culture

L929 cells were cultured in DMEM with high glucose (GE healthcare) complemented with 10% fetal bovine serum (Biological Industries, Israel), 2mM L-glutamine (Amresco, USA) and 1% antibiotics (100U/ml penicillin and 100 μg/ml streptomycin, Gibco, USA). The cells were maintained in a humidified conditions at 37 °C with 5% carbon dioxide incubator.

### LTP treatment procedure

Before LTP treatment, L929 cell suspension was seeded on 96-well plate with 200 μl per well at a concentration of 10^5^ cells/ml, and then kept in carbon dioxide incubator overnight with the same conditions described before. Besides, for treatment with NAC (Beyotime, China) and plasma, 5 mM of NAC was added to the wells 4 hours prior to exposure to plasma. According to a series of preliminary experiments, we determined LTP exposure time to be 5 s, 10 s, 15 s, 20 s, 25 s and 30 s.

### Cell viability assay

Cell viability was determined with MTT assay. After LTP-treated cells were cultured for 44 h, 20 μL MTT (Sigma, USA) was added into each well, and the cells were incubated for another 4 h. The culture medium was discarded, and formazan crystals were dissolved into dimethyl sulphoxide (Sigma, USA). The A570 absorbance values were determined on microplate reader (Tecan Spectra, Austria).

### Flow Cytometry analysis of cell cycle

After LTP-treated L929 cells were cultured for 48 h, all cultured medium is collected, and cells are digested with 0.25% trypsin (HyClone) without EDTA for 3 min. The digested cells and collected medium were centrifuged. The cells were washed with cold PBS twice and then fixed in 70% cold ethanol at least for 4 h. After incubation, cells were stained with propidium iodide (containing RNase1, Sigma, USA)), and then incubated in the dark at room temperature for 30 min. The samples were analyzed for DNA content profile with flow cytometry (Becton Dickinson, USA). The percentage of cells in G0/G1, S, and G2/M phases were assessed by ModFit LT software (Becton Dickinson, USA).

### Western blotting

Protein samples were extracted with RIPA-buffer (Heart, China) from LTP-treated cells after 24 h in culture, then heated at 95 °C °C for 5 min with sample buffer (250 mM Tris-HCl, pH 6.8, 10% glycerol, 4% sodium dodecyl sulfate, 2% β-mercaptoethanol, and 0.003% bromophenol blue). Proteins were separated on 12% SDS-PAGE, and electrophoretically transferred onto polyvinylidene difluoride (PVDF) membrane (Millipore, USA), and then blocked with 5% dried skim milk in Tris buffer saline with 0.1% Tween-20 (TBST) for 2 h at room temperature. The blocked membranes are rinsed three times in TBST for 10 min, and then incubated in the primary antibodies (anti-phospho p65, 1:5000, Abcam; anti-NF-κB P65 and anti-NF-κBIα Polyclonal Antibody, 1:2000, ABclonal; anti-cyclinD1, 1:200, Santa Cruz; anti-β-actin, 1:2000, CWbio) overnight at 4 °C. After being washed three times with TBST, the secondary antibody horseradish peroxidase (CWbio) was incubated on the membrane for 2 h at room temperature. Then the PVDF membranes were washed three times with TBST and incubated with ECL Plus Reagent (Millipore, USA) and scanned using the chemiluminescence (BioRad, USA). Immunoreactive products were quantified by image-Pro plus 6.0 software to determine the optical density of protein bands.

### Immunofluorescence

LTP-treated cells in 96 well-plate were washed three times with PBS, and then fixed by 4% paraformaldehyde for 10 min, blocked with 10% goat serum at room temperature for 1 h, and incubated with anti-phospho-p65 antibody (1:50, Abcam) overnight. The primary antibody was removed and washed three times with PBS, incubated with CY3-labeled secondary antibody (Beyotime, China) for 1 h. Nuclei were labeled with 1 μg/ml DAPI (Roche, Swiss) for 5 min and washed twice with PBS. Cells were visualized with inverted fluorescence microscope (Nikon, Japan).

### RNA interference of p65 expression

siRNA oligo was synthesized by Biomics Biotechnology (Jiangsu, China). p65-siRNA target sequences were: sense, 5′CUGAAGCUAUAACUCGCCUdTdT 3′, antisense 5′AGGCGAGUUAUAGCUUCAGdTdT3′. negative control sense: sense, 5′UUCUCCGAACGUGUCACGUdTdT3′, antisense 5′ACGUGACACGUUCGGAGAAdTdT3′. 500 μl DMEM high glucose medium without antibiotics contained 10^5^ L929 cells, which were inoculated in each well of a 24-well-plate, and then incubated in the conditions described before until they grew to 50% confluence. Before transfection, 2 μl 20 μM siRNA and 1.5 μl Lipofectamine^TM^ 2000 (Invitrogen, USA) were diluted with 48 μl and 48.5 μl Opti-MEM (Gibco, USA) respectively. The two reagents were mixed together and incubated at room temperature for 20 min, then added into each well. The final concentration of siRNA and Lipofectamine^TM^ 2000 were 67 nM and 2.5 μl/ml, respectively.

### Reverse transcription polymerase chain reaction (RT-PCR)

Total RNA was extracted by TRIzol reagent (Invitrogen, USA) from p65SiRNA-transfected cells after 24 h. RNA was reverse transcribed with M-MLV first strand cDNA Synthesis Kit (Omega, USA), amplified with a Taq-PCR master mix kit (Runde, China) and a pair of primer sequences as followed: sense 5′ TGTGGAGATCATCGAACAGCCG3′, antisense 5′ TTCCTGGTCCTGTGTAGCCATTGAT3′. Total RNA was reverse transcribed for 60 min at 37 °C and denatured at 85 °C for 5 min. PCR amplification is reacted in a total reaction volume of 25 μL with the following thermal cycling parameters: 94 °C for 3 min, 94 °C for 30 s, 55 °C for 30 s, and 72 °C for 1 min, for 30 cycles with the above three steps, and finally the last step of 72 °C for 5 min is performed. The house-keeping gene β-actin was amplified as an internal control with the primers: sense 5′CAACCTCTCgTACATCgg3′, antisense 5′ggTCTggTgCTCAAAAgg3′. PCR products were separated on 2% agarose gels and stained with ethidium bromide (Heart, China). DNA bands were visualized by UV transilluminator.

Quantitative Real-time RT-PCR was performed using SYBR Green I qPCR Mix (Takara) according to the manufacturer′s protocol. The primers sequences of cyclinD1 used in this study were as follows: sense 5′ GGAGCAGAAGTGCGAAGA 3′, antisense 5′ GAGGGTGGGTTGGAAATG 3′. The primers used for β-actin was same as those described above.

### Determination of intracellular ROS, superoxide anion (O_2_^−^) and nitric oxide (NO)

Intracellular ROS, O_2_^-^ and NO productions were estimated with 2′, 7′-dichlorofluorescin diacetate (DCFH-DA, Sigma, USA), dihydroethidium (DHE, beyotime, China), 3-Amino, 4-aminomethyl-2′, 7′-difluorescein, diacetate (DAF-FM DA, keygen biotech, China), respectively. LTP-treated cells were cultured for 24 h, and then washed with PBS for 3 times. After that, 200 μL 10 μM DCFH-DA diluted with DMEM (serum and phenol red free medium) was added into each well of the 96-well plate, incubated for 30 min at 37 °C in CO_2_ incubator, and then the cells were washed 3 times with PBS gently. ROS productions were visualized with inverted fluorescence microscope (Nikon, Japan). Fluorescence intensity was analyzed with image-Pro plus 6.0 software. The same steps was repeated for 200 μL 5 μM DAF-FM DA to detect NO. The cells treated with DHE were digested with 0.25% trypsin (HyClone) without EDTA for 3 min, and then collected and washed with cold PBS twice. After then the cells were resuspended in PBS and detected with flow cytometry (Becton Dickinson, USA).

### UV-visible absorption spectroscopy

Doublebeam UV-visible absorption spectroscopy (Hitachi, Japan) was used to detect nitrite/nitrate in the PBS and DMEM (without phenol red) treated by LTP. Spectra were recorded at room temperature in steps of 0.5 nm in the range from 200 to 500 nm using a 1 cm-path length quartz optical cell. Known concentrations of NaNO_2_ and NaNO_3_ were prepared in PBS.

### Detection of H_2_O_2_ in LTP treated PBS and DMEM

LTP induced H_2_O_2_ in PBS and DMEM were estimated with coumarin boronic acid (CBA) probe. PBS and DMEM with or without 5 mM CAT were added into 96-well plate with 200 μl per well and LTP exposure time were same as mentioned above. And then 2 μl CBA (0.1 M) was added into LTP treated PBS or DMEM, incubated for 30 min at room temperature. The fluorescence intensity of COH was detected with microplate reader (Tecan Spectra, Austria) at excitation and emission wavelengths are 355 nm and 460 nm. The concentration of H_2_O_2_ was quantified based on H_2_O_2_ standard curve.

### Statistics

The experiments of cell viability and cell cycle were repeated 3 times for each treatment dose. The values were expressed as means and standard deviation. All data were analyzed with one-way analysis of variance (ANOVA). All analyses were conducted with SPSS (vision 13.0). p  < 0.05 was the level of significance adopted in this study.

## Electronic supplementary material

Supplementary Information
