# Supplementary material for: Low temperature plasma promoting fibroblast proliferation by activating the NF-κB pathway and increasing cyclinD1 expression
Source: Sci Rep. 2017 Sep 15;7:11698. doi: 10.1038/s41598-017-12043-w (PMC5601921; doi:10.1038/s41598-017-12043-w)
Supplement: Supplementary file 1 — Supplementary Information [file 41598_2017_12043_MOESM1_ESM.doc]

Supplementary Information

Low temperature plasma promoting fibroblast proliferation by activating the NF-κB pathway and increasing cyclinD1 expression


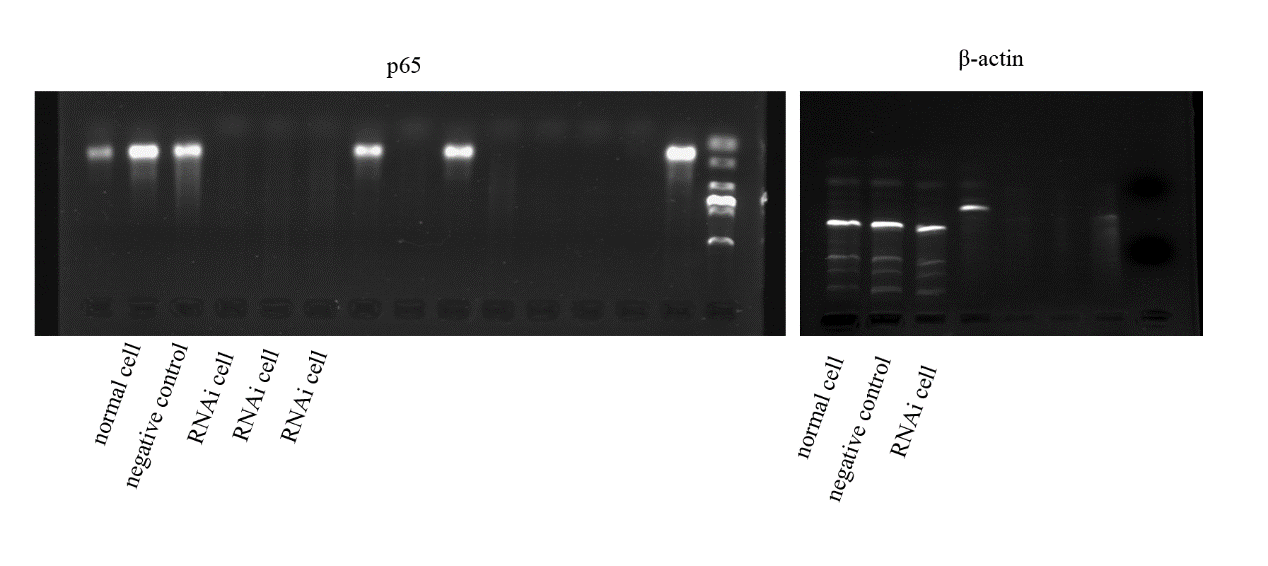


Figure 1. Effects of RNA interference on the mRNA transcription of p65. The full-length images of p65 mRNA transcription from reverse transcriptional PCR.


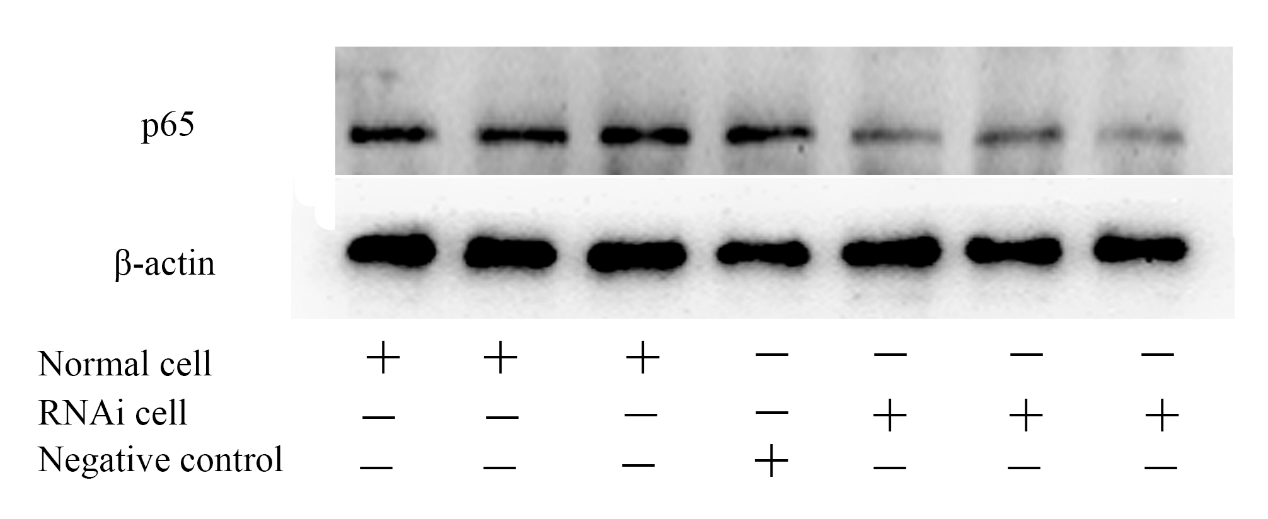


Figure 2. Effects of RNA interference on the protein expressions of p65. The full-length images of protein expression from western blot assays.


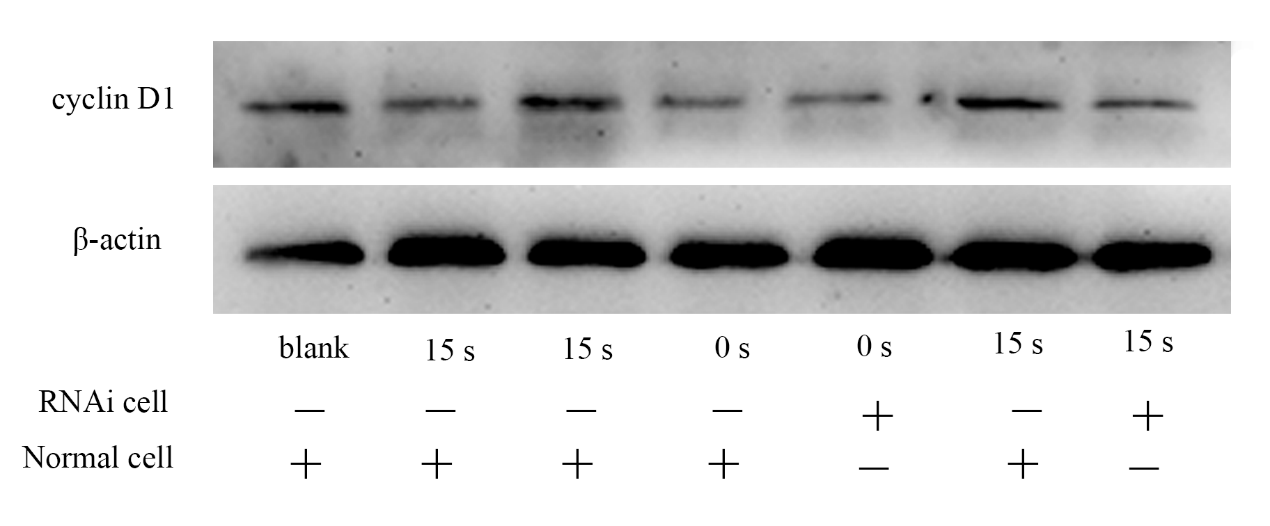


Figure 3. Effects of LTP on the protein expressions of CyclinD1. The full-length images of protein expression from western blot assays.
